# AntiHalluciNet: A Potential Auditing Tool of the Behavior of Deep Learning Denoising Models in Low-Dose Computed Tomography

**DOI:** 10.3390/diagnostics14010096

**Published:** 2023-12-31

**Authors:** Chulkyun Ahn, Jong Hyo Kim

**Affiliations:** 1 Department of Transdisciplinary Studies, Program in Biomedical Radiation Sciences, Graduate School of Convergence Science and Technology, Seoul National University, Seoul 08826, Republic of Korea; ahnck@snu.ac.kr; 2 ClariPi Research, ClariPi, Seoul 03088, Republic of Korea; 3 Department of Applied Bioengineering, Graduate School of Convergence Science and Technology, Seoul National University, Seoul 08826, Republic of Korea; 4 Department of Radiology, Seoul National University College of Medicine, Seoul 03080, Republic of Korea; 5 Department of Radiology, Seoul National University Hospital, Seoul 03080, Republic of Korea; 6 Center for Medical-IT Convergence Technology Research, Advanced Institutes of Convergence Technology, Suwon-si 16229, Republic of Korea

**Keywords:** computed tomography, deep learning, denoising, AI hallucination, image quality

## Abstract

Gaining the ability to audit the behavior of deep learning (DL) denoising models is of crucial importance to prevent potential hallucinations and adversarial clinical consequences. We present a preliminary version of AntiHalluciNet, which is designed to predict spurious structural components embedded in the residual noise from DL denoising models in low-dose CT and assess its feasibility for auditing the behavior of DL denoising models. We created a paired set of structure-embedded and pure noise images and trained AntiHalluciNet to predict spurious structures in the structure-embedded noise images. The performance of AntiHalluciNet was evaluated by using a newly devised residual structure index (RSI), which represents the prediction confidence based on the presence of structural components in the residual noise image. We also evaluated whether AntiHalluciNet could assess the image fidelity of a denoised image by using only a noise component instead of measuring the SSIM, which requires both reference and test images. Then, we explored the potential of AntiHalluciNet for auditing the behavior of DL denoising models. AntiHalluciNet was applied to three DL denoising models (two pre-trained models, RED-CNN and CTformer, and a commercial software, ClariCT.AI [version 1.2.3]), and whether AntiHalluciNet could discriminate between the noise purity performances of DL denoising models was assessed. AntiHalluciNet demonstrated an excellent performance in predicting the presence of structural components. The RSI values for the structural-embedded and pure noise images measured using the 50% low-dose dataset were 0.57 ± 31 and 0.02 ± 0.02, respectively, showing a substantial difference with a *p*-value < 0.0001. The AntiHalluciNet-derived RSI could differentiate between the quality of the degraded denoised images, with measurement values of 0.27, 0.41, 0.48, and 0.52 for the 25%, 50%, 75%, and 100% mixing rates of the degradation component, which showed a higher differentiation potential compared with the SSIM values of 0.9603, 0.9579, 0.9490, and 0.9333. The RSI measurements from the residual images of the three DL denoising models showed a distinct distribution, being 0.28 ± 0.06, 0.21 ± 0.06, and 0.15 ± 0.03 for RED-CNN, CTformer, and ClariCT.AI, respectively. AntiHalluciNet has the potential to predict the structural components embedded in the residual noise from DL denoising models in low-dose CT. With AntiHalluciNet, it is feasible to audit the performance and behavior of DL denoising models in clinical environments where only residual noise images are available.

## 1. Introduction

Computed tomography (CT) has established its role as an imaging modality of standard care in many clinical applications, providing clinicians with a detailed cross-sectional view of the human body’s internal structures [1,2]. Despite its irrefutable benefits, one persistent challenge in CT imaging is the trade-off between maintaining low radiation doses for patient safety and ensuring high-quality image acquisition [3,4]. Minimizing radiation exposure often results in images riddled with noise, which can potentially mask vital clinical details, thus diminishing the diagnostic value of the scan [5,6].

Recent years have seen revolutionary advancements in deep learning technology in medical imaging, especially for DL denoising in low-dose CT. Numerous studies have shown that DL denoising algorithms outperform the conventional iterative reconstruction techniques and can make images clearer and more suitable for diagnostic purposes [7,8,9,10,11,12]. Such advancements have undeniably positioned deep learning as an invaluable tool for CT denoising.

However, as is often the case with pioneering technologies, the use of deep learning in CT denoising is not devoid of challenges. It is suspected that DL models could induce hallucinations due to the possibility of unpredictable behavior, especially during noise inferencing in cases with a rare anomaly pattern. Indeed, recent studies showed that some DL applications might introduce artifacts or spurious details that are not present in the original scan during the image formation process. These inaccuracies, termed ‘hallucinations’, can mislead radiologists, resulting in potential misdiagnoses or erroneous clinical decisions [13,14,15,16].

Furthermore, evaluating the efficacy of deep learning-based CT denoising involves unique challenges. In traditional CT imaging, image quality assessment (IQA) typically follows the American Association of Physicists in Medicine task group report-233 (AAPM TG 233) guideline, using specialized phantoms to assess image quality [17]. This standard approach includes evaluating image noise, texture, task-based spatial resolution, and the detectability index. However, these methods are built upon the assumptions that CT imaging and denoising systems are linear and that their image quality can be evaluated in a straightforward manner; therefore, they do not provide capabilities to oversee the non-linear and complex nature of deep learning-based denoising techniques.

On the other hand, some studies accessed the quality of denoised medical images by relying on IQA metrics from the natural image processing domain [18,19,20]. These metrics fall into two categories: full-reference IQA (FR-IQA) and non-reference IQA (NR-IQA). Typical FR-IQA metrics include the peak signal-to-noise ratio (PSNR) and structural similarity index measure (SSIM) [21]. A major limitation of FR-IQA is that it necessitates paired low-quality and high-quality images from the same subject, which is mostly not possible in real clinical environments. In contrast, NR-IQA provides image quality metrics from a single input image and does not require a paired image set. Frequently used NR-IQA metrics include the natural image quality evaluator (NIQE) [22], blind/referenceless image spatial quality evaluator (BRISQUE) [23], and perception-based image quality evaluator (PIQE) [24]. However, these metrics only assess how perceptual and natural the images appear, and therefore, it is unclear whether these metrics can represent the image fidelity characteristics required to assess the acceptability of denoised medical images for use in imaging diagnosis. Therefore, there might be blind spots in conventional IQA metrics that are crucial for accessing image fidelity of DL-denoised images, as well as oversights related to the potential risk of AI-induced hallucinations we seek to mitigate.

Addressing these challenges requires a novel and comprehensive approach. One promising alternative is to focus on analyzing the residual noise. The residual noise, which is the difference between the original image and the denoised image, can enable a new and realistic control in identifying the behavior of DL denoising models. This is predicated on the understanding that successful denoising should not introduce structural components into the residual noise. By examining the residual noise and guaranteeing the absence of spurious structures, we can greatly diminish the chances of introducing hallucinations and ensure more reliable denoising outcomes.

In this paper, we embark on the development of a novel deep learning-based model designed to predict structures within the residual noise from CT denoising, which we named AntiHalluciNet. We evaluate the performance of AntiHalluciNet in predicting the spurious structures embedded in noise images at different noise levels, as well as its ability to assess the image fidelity of a denoised image by using only a noise component. We compare the image fidelity metric from AntiHalluciNet with those from FR-IQA and NR-IQA. We also explore the possibility of auditing the behavior of DL denoising models, such as by discriminating between the noise purity performances of different DL denoising models.

## 2. Materials and Methods

**Figure 1:**
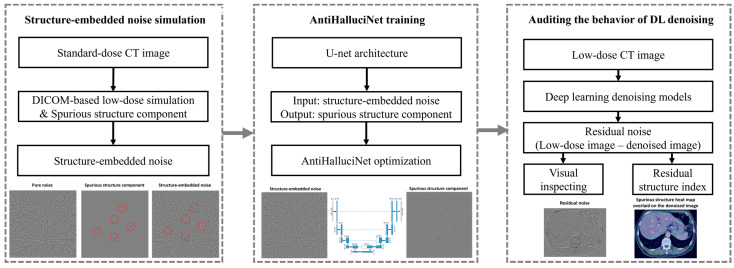
A flow diagram of AntiHalluciNet: structure-embedded noise simulation, AntiHalluciNet training, and auditing the behavior of DL denoising models.

Figure 1 presents a procedural flowchart outlining the steps involved in assessing DL denoising models with our AntiHalluciNet. In the first step, we simulate structure-embedded noise by combining DICOM-based low-dose simulation with artificially introduced spurious structural components using standard-dose CT images. The subsequent step involves training AntiHalluciNet on the simulated structure-embedded noise images using a U-net architecture. The structure-embedded noise serves as the input, while the spurious structure represents the output. The final step involves applying the trained AntiHalluciNet to audit the behavior of various DL denoising models. This is conducted by processing low-dose CT images with DL denoising models to obtain the residual noise (the difference between low-dose and denoised images). AntiHalluciNet then steps in to conduct a visual inspection and calculate a residual structure index, a metric devised to quantify the potential distortions or hallucinations.

### 2.1. Generation of Paired Structure-Embedded and Pure Noise Images

Using a total of 160 abdominal CT cases, we created a paired set of pure noise images and spurious structural component images. Pure noise images were generated using a realistic CT noise simulation algorithm, and the spurious structural components were generated by applying random circular masks to the high-frequency components of a CT image [25,26]. Then, structure-embedded noise images were generated by adding those spurious structures to the pure noise images.

#### 2.1.1. Base Dataset

A total of 160 patients’ contrast-enhanced liver CT scans were retrospectively collected for use as a base dataset. The CT scans were acquired using four CT scanners: Scanner 1 (GE Discovery CT750 HD, GE Healthcare, Milwaukee, WI, USA), Scanner 2 (Ingenuity CT, Philips Healthcare, Cleveland, OH, USA), Scanner 3 (SOMATOM Definition Flash, Siemens Healthineers, Erlangen, Germany), and Scanner 4 (Aquilion ONE, Canon Medical Systems, Otawara, Japan). All CT images were reconstructed with the filtered-back-projection (FBP) algorithm. Details about the CT scan parameters are shown in Table 1.

#### 2.1.2. Generation of Simulated Low-Dose CT

We employed a realistic low-dose simulation technique to generate low-dose CT images of the base dataset. Three sets of low-dose CT images were generated that had reduced-dose conditions at 75%, 50%, and 25% relative to that of the base data. This methodology allows for the direct application to DICOM CT images without the necessity for raw sinogram data or specialized reconstruction systems. It facilitates the generation of realistic noise patterns characteristic of low-dose CT, leveraging a mathematical model that encapsulates physical quantum statistics and the CT reconstruction procedure [26]. Previous studies have attested to the realism of this approach, particularly under reduced-dose conditions, in terms of noise magnitude and textural representation [27,28,29].

#### 2.1.3. Generation of Pure Noise Images

Mathematically, a simulated low-dose CT image, *y*, can be treated as the sum of a standard-dose CT image, *x*, and an additive noise component, *n*:

(1) y=x+n

From this relationship, the pure CT noise image, *n*, can be derived by subtracting the standard dose CT image, *x*, from the simulated low-dose CT images, *y*. As a result, three sets of pure CT noise images were derived with varying noise strengths corresponding to the reduced-dose conditions of 75%, 50%, and 25%.

#### 2.1.4. Generation of Structure-Embedded Noise Images

**Figure 2:**
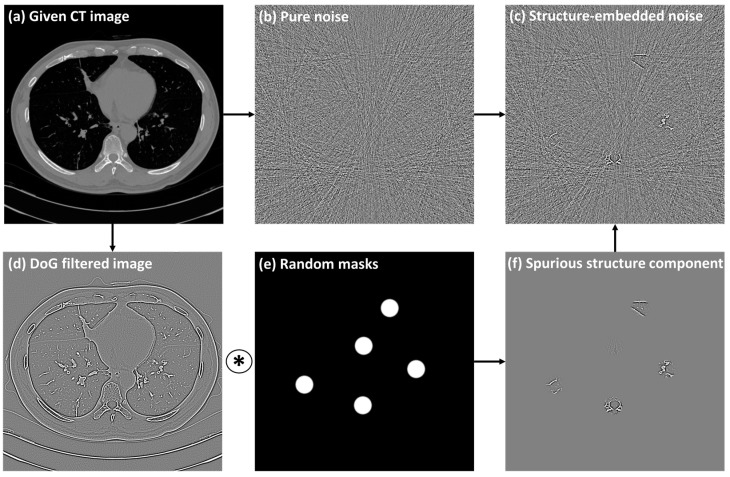
An example procedure of creating structure-embedded noise image. A pure noise image (**b**) was creating by applying a low-dose simulation technique to (**a**) the given CT image. High frequency component image (**d**) obtained by *DoG* filtering was multiplied by randomly placed circular masks (**e**) to produce spurious structural components (**f**). Then, the structure-embedded noise image (**c**) was obtained by combining the simulated pure noise images and structural components.

Figure 2 illustrates the procedure of creating structure-embedded noise images. This study used high-frequency components of a CT image as the structural components. The given CT image was subjected to Difference-of-Gaussian filtering with random sigma values ranging from 0.5 to 1.5 pixels. Then, the spurious structural components were produced by performing element-wise multiplication with randomly paced circular masks. This process is mathematically represented as:

(2) s=M∗ (DoG(x,σ))

wherein *DoG* denotes the Difference-of-Gaussian filtering operation, *σ* corresponds to the sigma value, *M* represents the random circular masks, ∗ is the element-wise multiplication, and *s* is the resultant spurious structural component. We set the diameter of the circular masks as 3 cm and placed them at five random locations.

Subsequently, to create the structure-embedded noise image *z*, the obtained spurious structural component *s* is superimposed onto the simulated pure noise image *n* as:

(3) z=n+s

### 2.2. AntiHalluciNet

We devised AntiHalluciNet, based on a modified U-net framework, to carry out the dedicated task of predicting the structural components in residual noise images [30]. While the traditional U-net consists of an encoding path, a decoding path, and a concatenation path, our modified U-net incorporated four max-pooling and up-pooling layers. Starting with 32 filters, we doubled the number of filters at each subsequent pooling layer, excluding a batch normalization layer.

#### 2.2.1. Training

We trained AntiHalluciNet to possess the ability to selectively predict spurious structural components in structure-embedded noise images. We took a supervised training approach and fed the network with structure-embedded noise images as the input and corresponding structural components as the ground truth. The network weights $\hat{\theta }$ are optimized using the Adam optimizer and an L1 loss function [31] so that

(4) $$\hat{\theta }\;\leftarrow \;arg\;\underset{\theta }{min}\frac{1}{N}\;\overset{N}{\underset{i=1}{\sum }}\;\left|s^{i}-f\left(z^{i};\;\theta \right)\right|$$

where *N* is the number of training images, and *f* is the deep learning model with network weights θ.

#### 2.2.2. Residual Structure Index

The residual structure index (RSI) is a novel index we devised to evaluate the potential distortions or hallucinations of image-denoising processes. It operates on the principle that residual noise—the difference between an original and a denoised image—can indicate the structural integrity of the denoised image. Essentially, if the residual noise contains structural components, this suggests that the denoised image may have introduced hallucinations or spurious details.

To calculate the RSI, we first process the residual noise through AntiHalluciNet, which predicts the structural components within it. We then quantify these predictions by taking the absolute value of the predicted structural component, averaging it, and dividing this average by the average absolute value of the residual noise. This calculation yields a value within the range of [0, 1]. To enhance the reliability of the RSI, we limit the calculation to the torso region of the image, excluding the background.

The RSI serves as an indicator of confidence of AntiHalluciNet in the presence of structural components relative to the residual noise. A higher RSI suggests a higher likelihood of spurious structural regions in the residual noise, implying potential distortions or hallucinations. Conversely, a lower RSI indicates the absence of such regions, suggesting a more successful denoising process that effectively eliminates noise while preserving the structural integrity of the image. This is mathematically articulated by the following equation:

(5) $$\mathrm{RSI}=\frac{\frac{1}{\left|M\right|}\sum_{\left(x,y\right)\in M}\left|\hat{s}\left(x,y\right)\right|}{\frac{1}{\left|M\right|}\sum_{\left(x,y\right)\in M}\left|\hat{n}\left(x,y\right)\right|}$$

where *M* denotes the morphology-based torso segmentation mask, and the calculations are conducted exclusively at the pixel coordinates (*x*, *y*) within this mask. Additionally, $\hat{s}$ represents the predicted structural component from the residual noise $\hat{n}$.

#### 2.2.3. Performance Verification

The performance of AntiHalluciNet in identifying spurious structures was verified by using the RSI along with a visual heatmap representation. The heatmap was created by systematically superimposing the absolute value of the predicted output onto the input residual image. The heatmap was intended to facilitate the visual inspection of the localization performance of AntiHalluciNet.

We employed a total of 20 validation cases, separate from the training dataset. Our approach comprised two key strategies. Firstly, we evaluated regions with known spurious structures, using ground truth masks to calculate the RSI and assess the model’s precision. Secondly, we investigated regions without spurious structures to understand the model’s behavior and reduce false positives. In these regions, we randomly positioned five circular masks, ensuring no overlap with the ground truth spurious structure areas, and computed the RSI.

#### 2.2.4. Performance Comparison with SSIM

For the further identification of spurious structures, we explored the potential of AntiHalluciNet in evaluating the overall image degradation, including image blurring and noise corruption. As reported in many previous studies, many conventional and DL-based denoising techniques are known to suffer from image blurring during the noise reduction process [32,33,34]. Previously, the SSIM was employed as a standard metric to evaluate the overall image degradation, but it has limited applicability due to the fact it needs a paired set of reference high-quality and degraded denoised images. In clinical reality, it is difficult to obtain such paired datasets in most cases. Therefore, we evaluated the potential of AntiHalluciNet in evaluating the overall image degradation using only the available residual noise image as a difference between the low-dose image and its degraded denoised counterpart.

We utilized the 20 validation cases to create three image sets: a reference set with high image quality taken to represent the reference dose condition; a low-dose set that underwent low-dose simulation at 50% of the reference dose condition; and a degraded denoised set, which had 30% less noise compared to the low-dose set with varying degrees of the image-blurring effect. With the degraded denoised set, we intended to mimic the imperfect denoising techniques where an image blurring effect was introduced during the denoising processing, as well as a situation where only a moderate degree of denoising was achieved. Here, we used Gaussian blurring with a sigma of 0.75 mm and mixing rates of 25%, 50%, 75%, and 100% to represent varying levels of the blurring effect being caused by a conventional denoiser.

We calculated the SSIM between the image pairs of the reference set and the degraded denoised set and used it as a reference measure of the image fidelity of the imperfect denoising technique. In addition, we measured the RSI of the residual images between the low-dose set and the degraded denoised set using AntiHalluciNet to consider its use as a surrogate measure for the image fidelity of the imperfect denoising technique. The SSIM and RSI values for the different degradation settings were compared to determine if the RSI could sensitively distinguish the image fidelities as compared to the SSIM.

### 2.3. Auditing the Behavior of DL Denoising Models

With AntiHalluciNet, we explored the potential of auditing the behavior of DL denoising models, including the prediction of hallucinations and evaluation of image degradation during denoise processing.

#### 2.3.1. DL Denoising Models

We employed three deep learning denoising models, including two publicly available pre-trained deep learning models [7,35] and the commercial deep learning-based software, ClariCT.AI (version 1.2.3, ClariPi, Republic of Korea) [36]. The first deep learning model was the Residual Encoder-Decoder CNN (RED-CNN). This model features a 10-layer architecture comprising 5 encoding convolution layers and an equivalent number of decoding deconvolution layers. The second model, Convolution-free Token2Token Dilated Vision Transformer (CTformer), was equipped with a tokenization block and a transformer block. Both models underwent training using the 2016 NIH-AAPM-Mayo Low Dose Grand Challenge dataset [37]. Contrastingly, the commercial software, ClariCT.AI, was engineered as a denoising solution built upon a U-net-based convolutional neural network. Its training paradigm involves the use of noise-added CT images as input, with the aim of predicting noise components as output, which is subsequently scaled and subtracted from the input noisy image to produce a denoised CT image.

#### 2.3.2. Real-World Evaluation Dataset

For the evaluation of the auditing of DL denoisers with AntiHalluciNet, we collected a real-world dataset of 30 patient CT scans consisting of paired standard-dose and low-dose images obtained simultaneously by using a split-dose technique. The split-dose scan was achieved by utilizing a 192-channel, third-generation dual-source CT scanner (SOMATOM Force, Siemens Healthineers, Erlangen, Germany) operated in dual-source mode with a fixed tube potential of 90 kVp, where the dose was divided between tube A (reference: 220 mAs, contributing 66.7% to the dose) and tube B (reference: 110 mAs, contributing 33.3% to the dose). For the standard-dose CT images, blended images from both tubes A and B were utilized, whereas for the low-dose CT images, only images from tube B were employed.

#### 2.3.3. Auditing of DL Denoisers

In exploring the potential of AntiHalluciNet for auditing the behaviors of DL denoisers, we considered two scenarios: inspecting the potential occurrence of hallucinations and monitoring the image fidelity to detect any potential defects in the denoiser causing abrupt image degradation.

For the first scenario, AntiHalluciNet-derived heat maps were generated for the residual images from the three DL denoisers, and the potential occurrence of hallucinations was visually inspected on the heat maps. For the second scenario, RSI values measured from the residual images of the DL denoisers were compared to determine whether AntiHalluciNet could detect the changes in the different DL denoisers during operation. We also calculated conventional IQA metrics, such as the typical FR-IQA of the SSIM and the NR-IQA metric of the NIQE. SSIM values were measured using the paired standard-dose and denoised images as a reference measure of image fidelity. NIQE values were measured on denoised images.

In this study, the calculation of two IQAs was conducted after normalizing the CT images to fit within a range of [0, 255]. This normalization process was based on a reference range from −160 HU to 240 HU, aligning with the standard abdominal window settings.

## 3. Results

### 3.1. Generation of Structure-Embedded and Pure Noise Images

**Figure 3:**
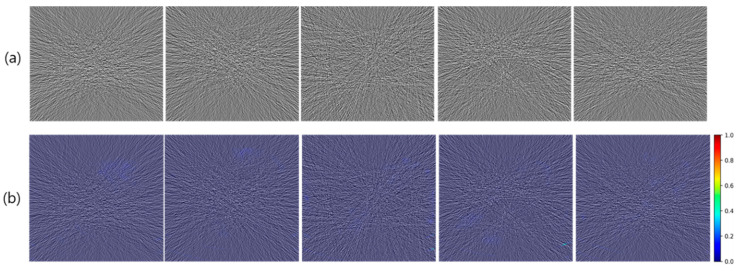
Selected examples of pure noise images (**a**) generated by using the experimental procedure described above and corresponding heatmaps (**b**) generated with AntiHalluciNet. Typical noise appearances are well represented in (**a**), and heatmaps with mostly clean backgrounds are shown in (**b**).

**Figure 4:**
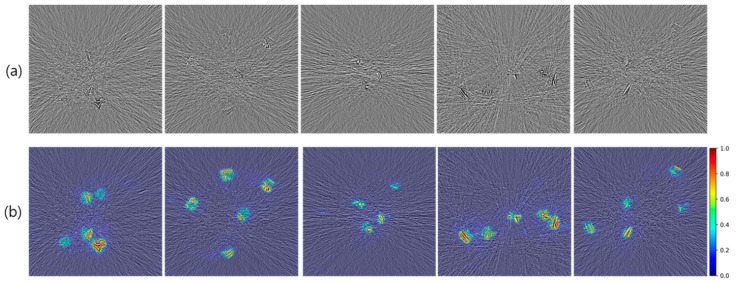
Selected examples of structure-embedded noise images (**a**) generated by using the experimental procedure described above and corresponding heatmaps (**b**) generated with AntiHalluciNet. Typical residual spurious structures are well represented in (**a**), and opaque colors are overlayed on the spots where spurious structures are located in (**b**).

Figure 3a and Figure 4a show selected examples of pure and structure-embedded noise images generated by using the experimental procedure described above. The pure noise images correspond to the noise strength of the 50% low-dose simulation, and the spurious structures are those derived using *DoG* filter with sigma values of 1.0 and 0.75 pixels.

In pure noise images, typical noise appearances are well represented, comprising streak patterns centering around the high attenuation objects as well as differing noise strengths depending on tissue attenuation levels. In structure-embedded noise images, simulated residual spurious structures are represented in an intermixed way along with background pure noises.

### 3.2. Verification of Prediction Performance with Heatmap

Figure 3b and Figure 4b show selected examples of the AntiHalluciNet-generated heatmaps overlayed on the example structure-embedded and pure noise images. As shown in the examples, the heatmaps have mostly clean backgrounds, while opaque colors are overlayed on the spots where spurious structures are located. These indicate the performance of AntiHalluciNet in highly specifically predicting the locations of spurious structures.

### 3.3. Verification of Prediction Performance with RSI Measurements

Table 2 shows the measured RSI values from the output of AntiHalluciNet on ROIs with embedded spurious structures, which are then compared with those on ROIs without structures. In all three low-dose simulation levels, the RSI values showed distinct differences between the ROIs with and without structures (0.83 vs. 0.03, 0.57 vs. 0.02, and 0.36 vs. 0.01 for 75%, 50%, and 25% dose simulation levels, respectively, with *p*-values < 0.001). It is noteworthy that the RSI value for the 75% dose simulation noise was much higher (0.83 vs. 0.36) than that of the 25% dose simulation noise. This indicates that AntiHalluciNet has higher confidence with regard to an embedded structure in a less noisy image than in a highly noisy image, which appears to resemble the behavior of human visual perception.

### 3.4. Performance Comparison with SSIM

Table 3 compares the mean SSIM and RSI values derived to indicate the fidelity of the degraded denoised images. The SSIM values were derived using the image pairs of the reference set and degraded denoised set, while the RSI values were derived without using the reference set. As shown in the table, the SSIM values gradually decreased from 0.9603 to 0.9333 as the mixing rate of the image blur component increased from 25% to 100%, whereas the RSI values increased more rapidly from 0.27 to 0.52 for the same mixing rates. This indicates that both the SSIM and RSI are able to distinguish the image fidelities of the degraded images from imperfect denoisers with different degradation settings, and there is a possibility that AntiHalluciNet could be more sensitive in the differentiation of image degradation levels.

### 3.5. Auditing of DL Denoisers

**Figure 5:**
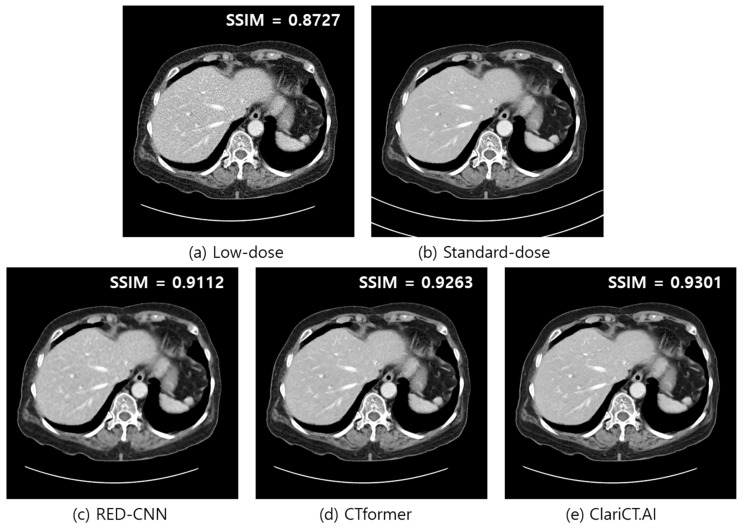
Comparison of low-dose image, standard-dose image, and denoised images from the three DL denoisers employed in this study. (**a**) Low-dose, (**b**) standard-dose, (**c**) RED-CNN, (**d**) CTformer, and (**e**) ClariCT.AI. The display window is [−160, 240] HU.

Figure 5 illustrates an example of denoised images generated by the three DL denoisers employed in our experiments. A low-dose abdomen image used for input, its standard-dose counterpart, and denoised images from RED-CNN, CTformer, and ClariCT.AI are compared. The image quality of the images from the three DL denoisers appears to be fairly good, and it is difficult to differentiate between them at first glance in a visual evaluation.

The SSIM values calculated using the paired standard-dose CT images showed slight differences among the three denoisers with 0.9112, 0.9263, and 0.9301 for the RED-CNN, CTformer, and ClariCT.AI, respectively, while that of the low-dose image was distinctly inferior at 0.8727.

**Figure 6:**
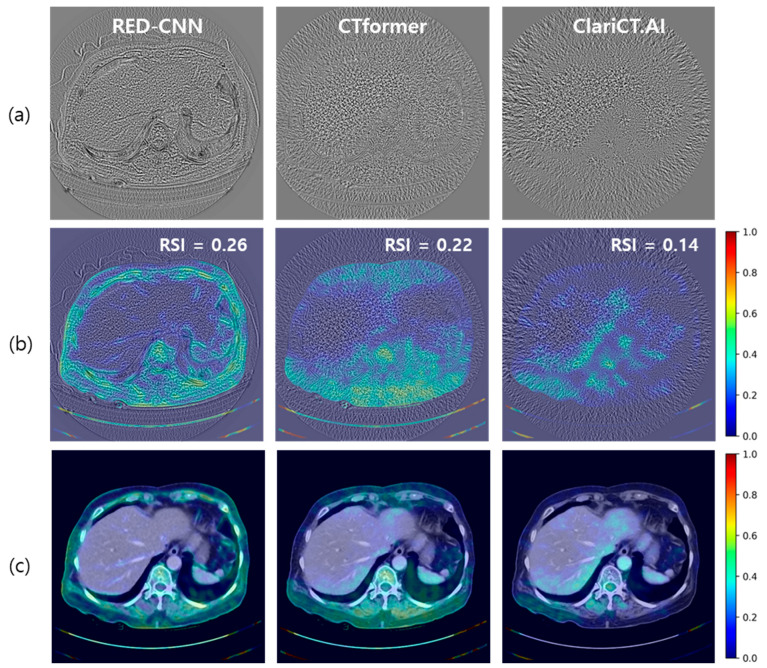
An example of (**a**) residual noise and (**b**) structure prediction heat maps (**c**) overlaid on denoised images from the three DL denoisers.

Figure 6a presents an example of residual noises from the three DL denoisers. Each residual noise was derived by subtracting the denoised image from the corresponding low-dose CT image. The structural components predicted by AntiHalluciNet were converted into heat map representations and were superimposed upon the denoised images in Figure 6b,c. It is evident that the AntiHalluciNet-derived heat map facilitates a more comprehensive visual inspection to identify the potential occurrences of hallucinations. The RSI values from AntiHalluciNet were 0.26, 0.22, and 0.14 for RED-CNN, CTformer, and ClariCT.AI, respectively, showing more distinct differentiation compared with the SSIM values.

Table 4 presents a comparison of the RSI with the typical FR-IQA of the SSIM and NR-IQA of the NIQE across three different denoisers—RED-CNN, CTformer, and ClariCT.AI. The SSIM mean values exhibit only marginal differences among the denoisers (RED-CNN (0.8725), CTformer (0.8916), and ClariCT.AI (0.9005)). On the contrary, more distinct distributions are observed in the RSI mean values, which were 0.28 for RED-CNN, 0.21 for CTformer, and 0.15 for ClariCT.AI. Notably, the SSIM values were in increasing order, while the RSI values were in decreasing order. The NIQE values, however, revealed a discrepancy in the performance order of denoising models: RED-CNN was 9.58, CTformer was 9.93, and ClariCT.AI was 9.06.

## 4. Discussion

While we have experienced the remarkable potentials of DL denoising models in recent years with regard to their ability to improve the image quality in low-dose CT, suspicions that DL models could cause hallucinations due to the possibility of unpredictable behavior also arose, especially during noise inferencing in cases with a rare anomaly pattern [13,14,15]. Therefore, gaining the ability to audit the behavior of DL denoising models is crucial in order to further advance the quality improvement cycle in clinical environments, thereby widening clinical applications. In this study, we presented a preliminary version of AntiHalluciNet, which is designed to predict the structural components embedded with the residual noise from DL denoising models in low-dose CT. With AntiHalluciNet, we explored the possibility of auditing the behavior of DL denoising models in clinical environments where only residual noise images are available.

Throughout our investigation, a significant emphasis was placed on inspecting the integrity of anatomical and pathological structures in the denoised images to compare how different DL denoising models perform against each other in this respect.

One of the most notable findings from our results was the relationship between the AntiHalluciNet-derived RSI and SSIM. Given our initial hypothesis that efficient denoising should not introduce structural components in the residual noise, we expected a high SSIM value, reflecting a retained image structure post-denoising, to align with a lower RSI value, signifying fewer structural components in the residual noise. Our empirical findings largely validated this, marking a step forward in assessing denoising methods beyond mere visual assessments and toward more concrete, quantifiable metrics.

For comparison purposes, we incorporated a frequently used NR-IQA metric, the NIQE, which does not require a paired image set. Unlike the SSIM, the NIQE values revealed a discrepancy in the performance order of denoising models. In both the RSI and SSIM, the performance order of the three denoising models was RED-CNN, CTformer, and ClariCT.AI in increasing order. However, in the NIQE, the orders were switched between CTformer and RED-CNN. Based on our understanding that the SSIM is an established reference method for image quality assessment, our results indicate that the NIQE as an IQA metric does not exhibit sufficient reliability for the purpose of evaluating DL denoisers. These findings reassure that the AntiHalluciNet-derived RSI can be the most reliable and practical image quality metric, which can be accessed using only low-dose images.

Furthermore, our research underscores the indispensable role of visual inspection in denoising, in which AntiHalluciNet assists in generating heatmaps from structural components of residual noise, superimposing them onto denoised images for clear image evaluation. This method provides an intuitive assessment to identify potential distortions or alterations, as depicted in Figure 6.

This study offers significant insights into the AI-aided inspection of the image denoising process in low-dose CT imaging but presents a few limitations. We employed a DICOM-based CT simulation technique for generating pure noise images under different low-dose scan settings and generated structure-embedded images by simply adding the spurious structural components to the generated pure noise. This might not be universally representative, thereby influencing our evaluation metrics. These constraints should be considered when interpreting our findings and extrapolating them to broader contexts. Our dataset also lacked images specific to various pathologic medical conditions, potentially affecting real-world clinical applicability. Also, in our real-world dataset, we exclusively utilized a CT scanner from a single vendor, which may limit the universality of our results across different manufacturers. On the other hand, this study also has limitations in terms of the metric used for comparison. We employed a publicly available NIQE algorithm, which was fitted for the IQA purpose of assessing natural images. Although the NIQE algorithm has been known to be extended for use with medical images, it would require detailed knowledge and well-designed data engineering work, which is regarded as being out of the scope of this study. Therefore, the limitation of the NIQE shown in this study might be relevant to its extended version. Additionally, we employed only traditional IQA algorithms in comparison with our proposed RSI metric. There are a few studies that have used deep learning-based IQA (DL-IQA) methods, which were applied for the IQA of CT images [38,39,40]. However, these DL-IQA algorithms are not publicly available, limiting their application in our research.

Despite these limitations, our research unequivocally underscores the potential of the AI-aided inspection of DL denoise models for low-dose CT imaging. The challenges posed by denoiser-induced hallucinations are not to be minimized, especially with the advent of DL-based denoising techniques. Complex architectures employed in DL denoisers lead to difficulties in characterizing and predicting behaviors under diverse imaging conditions encountered in real-world clinical environments. We believe that this study paves the way towards the more robust and reliable auditing of DL denoising models by integrating a comprehensive AI-aided analysis of residual noise images. As the medical imaging field continues to evolve and integrate advanced AI techniques, understanding and addressing these challenges will be of paramount importance. Our study serves as a foundational step in this direction, emphasizing the need for continual innovation and scrutiny in the application of deep learning for medical imaging.

In conclusion, our developed AntiHalluciNet has the potential to predict the structural components embedded in the residual noise from DL denoising models in low-dose CT. With AntiHalluciNet, it is feasible to audit the performance and behavior of DL denoising models in clinical environments where only residual noise images are available.

## Figures and Tables

**Table 1:** Acquisition parameters of CT scans.

	Scanner 1	Scanner 2	Scanner 3	Scanner 4
Number of cases (Train/Val)	35/5	35/5	35/5	35/5
Tube voltage (kV)	120	100	100	100
Mean tube current (mAs)	135.4 ± 25.3	150.2 ± 24.7	149.7 ± 28.1	152.3 ± 30.6
Reconstruction kernel	Standard	B	B30f	FC08
Slice thickness (mm)	2.5 mm	3 mm	3 mm	3 mm

**Table 2:** Comparison of RSI measurements on ROIs with and without embedded structures.

	ROIs with Embedded Structure	ROIs without Structure	*p*-Value *
25% dose simulation	0.36 ± 0.29	0.01 ± 0.02	<0.001
50% dose simulation	0.57 ± 0.31	0.02 ± 0.02	<0.001
75% dose simulation	0.83 ± 0.25	0.03 ± 0.03	<0.001

* *p*-values were calculated with the Mann–Whitney U test, and *p* < 0.05 indicates a statistically significant difference.

**Table 3:** Comparison of RSI and SSIM values derived to indicate the fidelity of the degraded denoised images. While the SSIM values were derived using the paired reference set and degraded denoised image set, the RSI values were derived using only the residual noises of the degraded denoised set without using the reference set.

	SSIM ↑↑	RSI ↓↓
25% mixing	0.9333 ± 0.0133	0.27 ± 0.08
50% mixing	0.9490 ± 0.0130	0.41 ± 0.08
75% mixing	0.9579 ± 0.0146	0.48 ± 0.08
100% mixing	0.9603 ± 0.0169	0.52 ± 0.07

**Table 4:** Comparison of RSI with FR-IQA and NR-IQA metrics across three denoisers—RED-CNN, CTformer, and ClariCT.AI.

	RSI ↓↓	FR-IQA	NR-IQA
		SSIM ↑↑	NIQE ↓↓
RED-CNN	0.28 ± 0.06	0.8725 ± 0.0279	9.58 ± 0.32
CTformer	0.21 ± 0.06	0.8917 ± 0.0254	9.93 ± 0.30
ClariCT.AI	0.15 ± 0.03	0.9005 ± 0.0248	9.06 ± 0.27

## Data Availability

The datasets generated or analyzed during the study are available from the corresponding author upon reasonable request.
